# An origami paper-based electrochemical biosensing platform for quality control of agri-food waste in the valorization strategy

**DOI:** 10.1007/s00604-022-05392-5

**Published:** 2022-08-03

**Authors:** Noemi Colozza, Erika Di Meo, Angelica Mucaria, Danila Moscone, Fabiana Arduini

**Affiliations:** 1 Department of Chemical Science and Technologies, University of Rome “Tor Vergata”, Via della Ricerca Scientifica, 00133 Rome, Italy; 2 SENSE4MED S.R.L, Via Bitonto 139, 00133 Rome, Italy

**Keywords:** Glucosinolates, Myrosinase, Carbon black, Prussian Blue nanoparticles, Glucose oxidase, Chronoamperometry

## Abstract

The increasing demand for food and the need for a sustainability vision in the agri-food sector have boosted novel approaches for food management, enhancing the valorization of wastes and by-products belonging to the food industry. Herein, we present a novel paper-based origami device to assess the amount of both glucosinolate and glucose in a food waste product belonging to *Brassicaceae* plants, to evaluate the quality value and the correct management of waste samples. The device has been designed as an origami paper-based platform constituted of two paper-based biosensors to work synergistically in a multiplexed detection. In detail, a monoenzymatic biosensor and a bienzymatic biosensor were configured for the detection of glucose and glucosinolates, respectively, using filter paper pads preloaded with glucose oxidase and/or myrosinase. To complete the paper-based platform, the enzyme-preloaded pads were combined with office paper-based electrodes modified with Carbon black/Prussian Blue nanoparticles for the measurement of enzymatic by-product at a low applied potential (i.e., 0 V versus Ag/AgCl). Overall, this paper-based platform measured glucose and glucosinolate (i.e., sinigrin) with a linear range up to 2.5 and 1.5 mM, and detection limits of 0.05 and 0.07 mM, respectively. The repeatability corresponded to an RSD% equal to 5% by testing 10 mM of glucose, and 10% by testing 1 mM of sinigrin. The accuracy of the developed multiplex device was evaluated by recovery studies at two different levels of sinigrin, i.e., 0.25 and 0.5 mM, obtaining recoveries values equal to (111 ± 3) % and (86 ± 1) %, respectively. The multiplex detection of both glucose and glucosinolate in *Brassicaceae* samples evaluates the quality values of the waste sample, ensuring the quality of the re-used food product waste by using an eco-designed analytical tool. The combination of paper-based devices for quality control of food waste with the re-use of these food products represents a sustainable approach that perfectly matches sustainable agrifood practices as well as the overall approach of the circular economy.

**Graphical abstract:**

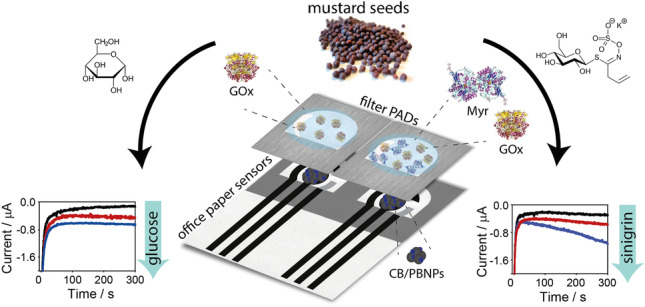

## Introduction

Among the effects of the progressive growth of the population, the over-production of agricultural waste is a global issue that arises as a collateral consequence of the increasing demand for food. The sustainable management of the food chain, from the agriculture field to the distribution of food and its storage, is a challenge for the modern world that requires specific efforts in all the steps involved. In addition, the production of food waste is a crucial issue, taking into account that the United Nations Environment Program in the report “Food Waste Index 2021” has estimated around 931 million tons of food waste in the world during 2019. The Food and Agriculture Organization of the United Nations (FAO) has warned since 2014 the global community regarding the great costs associated with food wastage: about 2.6 trillion USD are lost each year on the global level, in which are comprised 700 billion USD related to environmental costs and 900 billion USD related to social costs. In this framework, the waste coming from agriculture plays a significant contribution, representing over 30% of worldwide agricultural productivity. A further source of food waste comes from the industry of food processing, from which a variety of food by-products can be released, arising possible issues both in environmental and economic terms [1].

Among foods that are good candidates for possible recycling and re-using, the *Brassicaceae* family including cauliflower, horseradish, and mustard seeds, represents a predominant class for the presence of nutritional components [2–5]. For instance, glucosinolates present in *Brassicaceae* samples are a large group of sulfur-containing secondary metabolites with anti-inflammatory and anti-cancer properties [6]. However, the level of glucosinolates can largely vary in vegetable samples, depending on the species, the plant age, the plant part, and the practices followed for crop management [7].

In detail, glucosinates are present in the vacuole of plant cells [8], thus separated from the thioglucoside glucohydrolase enzyme, also known as myrosinase (Myr), which is in the cytoplasmic region [9]. When the plant cells are damaged, also during crop management, the plant releases glucosinolates in the cytoplasm which react with Myr [10], producing glucose and a variety of by-products (e.g., isothiocyanates and thiocyanates [11, 12]). However, the loss of these enzymatic by-products by volatilization [12, 13] decreases the food composition values.

To control the quality of *Brassicaceae* foodstuff and waste, the food composition values need to be assessed by determining the content of glucosinolates. Glucosinolates can be detected by a variety of laboratory-based analytical methods, including spectrophotometry [8, 10], gas chromatography-mass spectrometry [14, 15], high-performance liquid chromatography with UV detection [15, 16], high-performance liquid chromatography with tandem mass spectrometry [17], high-performance liquid chromatography coupled with a diode array detector and circular dichroism [18], reflectance spectroscopy [19], colorimetric sensor liquid array [20], and visible/near-infrared spectroscopy [21]. However, most of these techniques require a laboratory set-up, skilled personnel, high costs of instrumentation, long times of execution, and long sample treatment.

In recent years, analytical electrochemical techniques have gained great success, thanks to their great advantages, such as cost-effectiveness, easiness of use, miniaturization, and the suitability for carrying out in situ analyses. In the case of glucosinolate detection, some electrochemical (bio)sensors have been reported in the literature based on the exploitation of bi-enzymatic systems using Myr and glucose oxidase (GOx) enzymes as biocomponents. The advantage of using enzyme-based biosensors relies on the possibility of working in mild conditions, typically consisting of buffer solutions at close neutral pH and low applied potentials. In this regard, glucose detection can be achieved both by enzymatic biosensors [22–24] and non-enzymatic sensors [25–29] with competitive analytical performances. However, non-enzymatic sensors usually need for basic conditions (e.g., 0.1 M NaOH) and relatively high oxidative potential (e.g., > 0.45 V). On the other hand, a smart approach for glucose detection is based on the use of GOx coupled with Prussian Blue nanoparticles for the measurement of enzymatic by-product hydrogen peroxide at a low applied potential (e.g., 0.0 V vs Ag/AgCl) and under buffered condition (e.g., phosphate buffer, pH = 7.4) [22].

The bi-enzymatic strategy was exploited by Thurston’s group [30] to develop an amperometric biosensor based on the co-immobilization of both Myr and GOx tested in real samples of rapeseed. Alternatively, glucosinolates were determined in rapeseed by mean of a pH electrode where Myr enzyme was immobilized using a nylon membrane [31]. Another example of biosensors for detecting glucosinolates reported an optical assay suitable for both in situ analysis and remote control [12]. In other cases, an amperometric enzymatic method based on the flow injection technique was realized for the measurement of broccoli leaves, cauliflower, and sinapis seeds [32], and a bienzymatic biosensor using colloidal dispersion of gold and multi-walled carbon nanotubes braided with Teflon membrane to efficiently measure glucosinolates in a linear range between 0.02 and 1 mM [11].

In the last decades, the fabrication of electrochemical (bio)sensors has been progressively improved to answer the need for user-friendly, portable, low-cost, and non-polluting devices. The paper has been proved as an outstanding material that can provide these requirements and introduce more features [33, 34]. Intriguingly, by rationally selecting the paper type (i.e., different porosity) and by designing the number of paper layers used (i.e., by folding or overlapping the paper in multifarious formats), it is possible to realize paper-based devices with origami-like configurations ready/easy-to-use [34]. In detail, our group has demonstrated how the paper is well suited for the realization of electrochemical enzymatic biosensors for several applications such as environmental monitoring and diagnostics [35–39]. These studies have shown that the enzymatic activity can be preserved when the enzyme is loaded into a porous paper matrix, within delimited areas patterned by wax; the additional loading of the enzymatic substrate on the paper device allows for delivering ready-to-use biosensors.

Here we report an origami paper-based platform for multiplex detection of *Brassicaceae* composition values, constituted of:

(i) a bienzymatic paper-based origami biosensor for the detection of glucosinolates obtained combining office paper for the screen-printing of the electrochemical cell and filter paper for the loading of the enzymes, namely Myr and GOx;
(ii) a monoenzymatic paper-based origami biosensor for the detection of glucose obtained combining office paper for the screen-printing of the electrochemical cell and filter paper for the loading of GOx enzyme.

Thanks to the origami-like design, the device can be used by assembling the office paper sensor with the filter paper analytical device (PAD) through simple overlapping. For the measurement, a few microliters of sample are simply drop-cast onto the origami device, resulting in overall simplicity and safety of handling. To improve the electrochemical performances, the working electrode is modified with a dispersion of Carbon black-Prussian Blue nanoparticles (CB-PBNPs) for the detection of hydrogen peroxide at low applied potential (i.e., close to 0 V versus Ag/AgCl) [35, 36, 40].

The multiplex analysis obtained by measuring both glucose and glucosinolate allows for the evaluation of the quality of the target *Brassicaceae* sample. Indeed, the eventual hydrolysis of glucosinolates, with the consequent presence of enzymatic by-products such as isothiocyanates, makes the sample difficult to handle because of their volatility and odorous characteristics [13]. At the same time, the eventual loss of volatile by-products results in a lower quality value of the food for the decrease of its beneficial properties [13]. Importantly, the combined measurement of glucose and glucosinolates present in the sample allows for the correct quantification of glucosinolates, avoiding overestimation due to the physiological content of glucose (i.e., not produced by the Myr enzymatic reaction). This new concept of origami paper-based device has been challenged using Indian mustard seeds to demonstrate the suitability of sustainable analytical tools as smart biosensing systems for quality control of waste products, boosting food waste recycling.

## Experimental

### Reagents and equipment

All the reagents used were chosen of analytical grade. A phosphate buffer solution was prepared in distilled water using 50 mM KH_2_PO_4_ and 50 mM KCl, pH = 6.0, purchased from Carlo Erba. CB-PBNP powder was prepared using K_3_Fe(CN)_6_ and HCl 37% (w/w) obtained from Sigma Aldrich, and FeCl_3_ obtained from Fluka. CB N220 of industrial standard grade was obtained from Cabot Corporation (Italy). N, N-dimethylformamide (DMF) was from Merck Millipore. Glucose, sinigrin hydrate, and GOx enzyme were purchased from Sigma Aldrich. Myr enzyme of 38 U/mL, 50 U/mg of specific activity, and purification factor of 71 was kindly provided by Dr. Eleonora Pagnotta from the laboratory of the Council for Research in Agriculture and Agrarian Economy (CREA) placed in Bologna (Italy). Indian mustard, brown mustard seeds (*Brassica Juncea* L.) used as a real matrix sample were purchased from a local market. The chronoamperometric analyses were performed using the chronoamperometric technique performed by a portable MultiEmStat^3^ (PalmSens, Netherlands) at a fixed potential of 0.0 V for 300 s.

### Printing procedures

Home-produced screen-printed electrodes were realized using optimized protocols [35–39]. Firstly, a pattern was drawn using Adobe Illustrator software for the printing of wax on sheets of A4 dimensions of both the office paper (Copy 2, 80 g/m^2^, Fabriano, Italy) and the filter paper (67 g/m^2^, Cordenons, Italy), using a ColorQube 8580 Xerox printer. The pattern is used to realize a hydrophobic barrier in which the aqueous solution can be retained, avoiding its absorption by capillarity throughout the paper layer. The resulting hydrophilic areas on the office paper are used to print the electrochemical cell, while the hydrophilic areas on the filter paper delimit the sampling area where the enzymes are subsequently pre-loaded. The wax pattern is cured at 100 °C for 2 min to allow the wax to homogeneously permeate through the cellulose matrix.

The three-electrode cell was screen-printed using a 245 DEK (Weymouth, UK) serigraphic printer. The working and counter-electrodes were printed using a graphite-based ink (Electrodag 423 SS), while the pseudo-reference electrode was made by using Ag/AgCl-based ink (Electrodag 6033 SS), purchased from Henkel. The resulting working electrode geometric area is about 12.6 mm^2^ [36].

### Office paper sensor modification

Firstly, the working electrode was modified in 3 steps using each time 2 μL of CB-PBNP dispersion drop-cast on the electrode surface, for a total final volume of 6 μL, as optimized elsewhere [35, 36]. The CB-PBNP powder was prepared as follows [35–39]: (i) 1 g of CB was added to 10 mL of a solution of 0.1 M K_3_Fe(CN)_6_ in 10 mM HCl and kept under magnetic stirring for 10 min; (ii) afterward, 10 mL of a solution of FeCl_3_ in 10 mM HCl was added to the solution and kept under magnetic stirring for 10 min; (iii) thus, the dispersion was centrifuged for seven times, washing the precipitate with 0.1 M HCl until a clearer supernatant solution was obtained; (iv) finally, the precipitate was treated 90 min at 100 °C and then pounded in a marble mortar, resulting in a fine powder. The dispersion of CB-PBNPs was prepared using 10 mg of this powder dispersed into 10 mL of a mixture of DMF: distilled water 1:1 (v/v), to obtain a concentration equal to 1 mg/mL, followed by a sonication step of 60 min at a frequency of 59 kHz using an ultrasonic sonicator bath (Falc Instruments, Italy).

### Preparation of the mono/bienzymatic origami biosensor

In the case of the monoenzymatic origami biosensor, 4 μL of 100 U/mL GOx solution was pre-loaded on the hydrophilic sampling areas of the filter paper PADs and left to dry. In the case of the bienzymatic origami biosensor, after the pre-loading step with GOx, 8 μL of 38 U/mL Myr was further added on the same filter paper PADs and left to dry. In both cases, each dried PAD was then overlapped on an office paper sensor, previously modified with the CB-PBNP dispersion. The hydrophilic areas, delimited by the wax pattern, were carefully matched and fixed together by applying slight strips of paper tape on the wax pattern. The origami biosensors were then ready for the measurement, carried out by just drop-casting 40 μL of the sample solution to re-dissolve the pre-loaded enzyme(s). Because of the volume of the sample solution, the original concentrations of the pre-loaded enzyme(s) resulted to be diluted at 1:10 and 1:5 (v/v) for GOx and Myr, respectively, thus obtaining the final concentrations of 10 U/mL for GOx and 7.6 U/mL for Myr. The volumes and concentrations of the enzymes were optimized during this study, as described in “Results and discussion.” The response of the mono/bienzymatic origami biosensor is halved after 3 days when stored under RT conditions, while it is stable up to 14 days when stored under vacuum conditions.

### Real sample extraction

The extraction of glucosinolates from *Brassica juncea* L. samples was carried out by following the Doheny-Adams et al. [41] protocol with some modifications. In detail, 0.1 g of Indian mustard seeds were added in 50 mL of tap water and boiled for 4 h on a hot plate at a constant temperature of 100 °C, under magnetic stirring. For the recovery study, the same boiling steps were repeated by adding known amounts of standard sinigrin, namely 0.25 and 0.50 mM, used as the reference glucosinolate in this study. After boiling, the extracted solutions were centrifuged at 4000 rpm for 10 min, thus the supernatants were stored at 4 °C until usage.

## Results and discussion

### Working principle of the paper-based origami platform

In this work, we developed a paper-based origami platform with two different biosensors for the monitoring of the composition values in *Brassicaceae* extracts, namely: (i) a monoenzymatic biosensor for the detection of glucose, based on the quantification of hydrogen peroxide by GOx enzymatic reaction and (ii) a bienzymatic biosensor for the measurement of glucosinolates, by exploiting the combination of Myr and GOx enzymes.

**Scheme 1:**
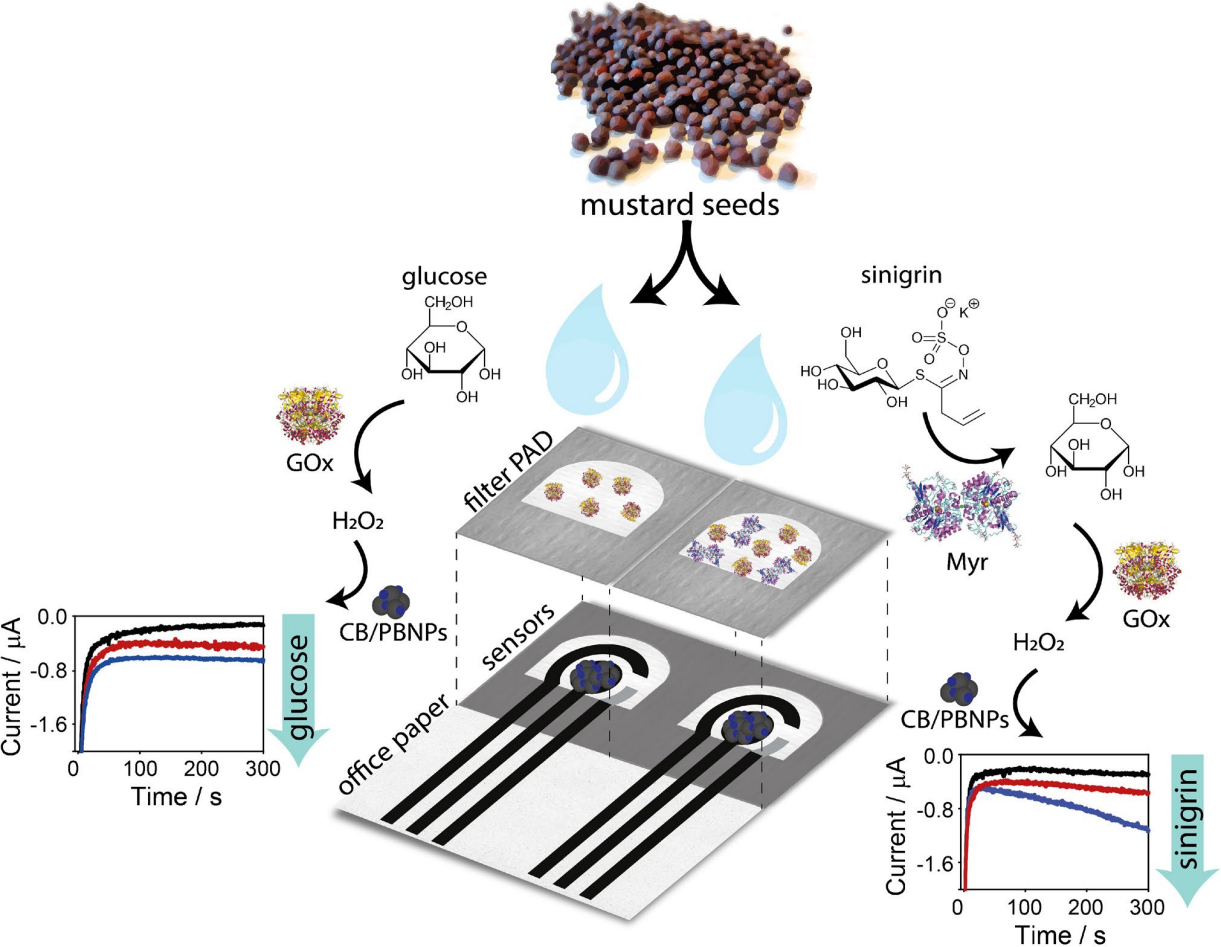
The working principle of the here developed paper-based origami platform for multiplex analyses of sinigrin and glucose

The key principle of this biosensor relies on the unique properties of the paper and the origami-like configuration of this device. Besides general advantages, such as the low cost and the easiness of disposal (e.g., incineration), the paper was chosen for high versatility to deliver a reagent-free and pump-free microfluidics device. In detail, our platform was built up by combining two types of paper, namely office paper and filter paper, to rationally exploit the different properties of these paper substrates as well as the vertical microfluidic (Scheme 1). On the one hand, the office paper was chosen as the substrate for electrode printing, thus realizing a three-electrode cell in the working area. This kind of paper is desirable for electrode printing with respect to plastic- or ceramic-based sensors because it can provide a suitable surface for the serigraphic deposition of the conductive inks and for the classical drop analysis, with the advantages of being more environmentally friendly and cost-effective [42].

On the other hand, the filter paper was chosen for entrapping and storing the selected reagents, thanks to its high porosity [33, 34]. As shown in previous studies [35–39], an enzyme solution can be pre-loaded on filter paper and left do dry, thus obtaining a ready-to-use PAD. Indeed, the enzyme is re-dissolved as soon as the sample is dropped on the PAD and the enzymatic reaction occurs, followed immediately after by the electrochemical one. In the present study, we aimed to exploit this strategy by pre-loading GOx or both the enzymes GOx and Myr on the filter paper, realizing a platform constituted of a monoenzymatic biosensor and a bienzymatic biosensor, ready to be applied when exposed to the enzymatic substrate (i.e., glucose or glucosinolates).

To accomplish this goal, the electrochemical cell screen-printed onto office paper and the enzyme- preloaded filter PADs were combined in an origami-like device with a vertical microfluidic configuration. When a solution is dropped, the reagents loaded on filter paper reached the underlying electrodes for electrochemical measurement, thanks to the diffusion through cellulose matrix of the filter PADs. In the case of the monoenzymatic biosensor, the reaction between the GOx and glucose will produce H_2_O_2_ detected at the office paper-based sensor. In the case of the bienzymatic biosensor, glucosinolates react with Myr, producing glucose, which in turn reacts with GOx producing H_2_O_2_. Herein, sinigrin was chosen as a model compound within the family of glucosinolates, because it is present in Indian mustard seeds (*Brassica juncea* L.), plant species chosen for real matrix analyses [43]. In both cases, H_2_O_2_ is detected at a low potential (i.e., 0.0 V versus Ag/AgCl pseudo-reference printed electrode), thanks to the electrocatalytic capacity of PBNPs, previously deposited onto the working electrode [35, 36, 44].

### Configuration of the monoenzymatic origami sensor

**Fig. 1:**
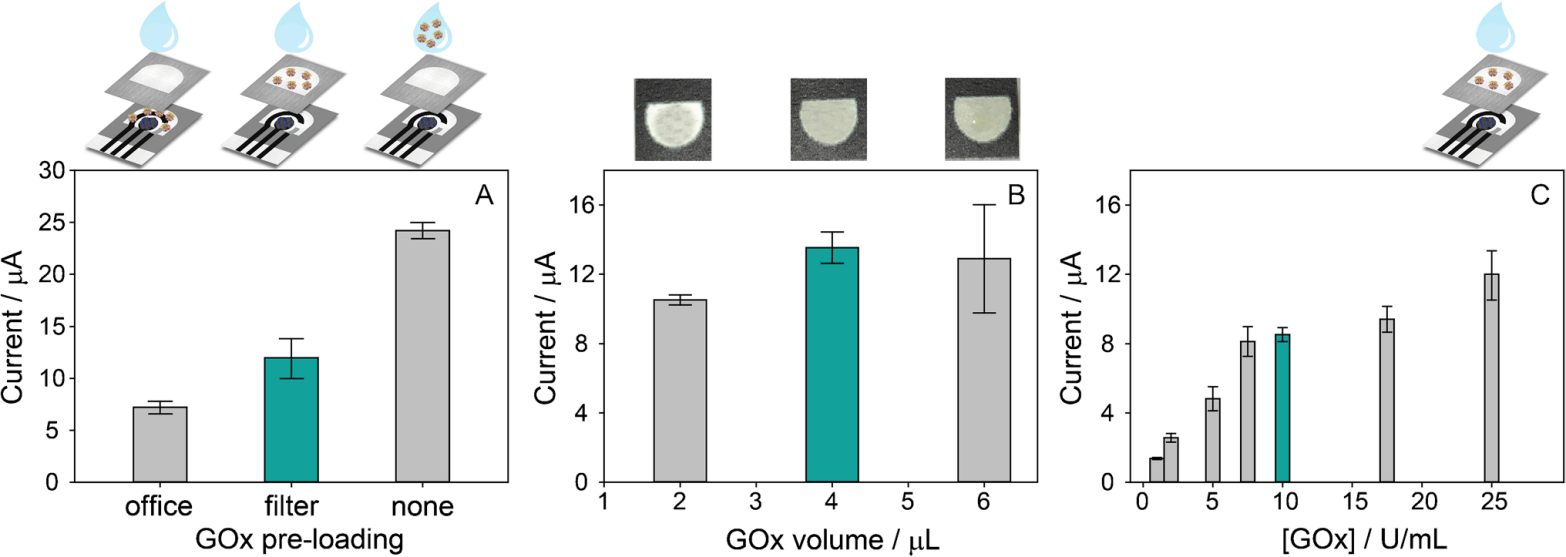
Chronoamperometric responses (*E* = 0.0 V, *t* = 300 s) obtained for the study of the GOx pre-loading step. (**A**) Comparison among 4 μL of GOx pre-loaded on the electrochemical cell area of the office paper sensor, 4 μL of GOx pre-loaded on the filter PAD, and GOx added to the sample solution; in all the cases, 250 U/mL GOx was used for the pre-loading, obtaining a final concentration of 25 U/mL when the sample solution was added. (**B**) Comparison among different volumes used to pre-load 250 U/mL GOx on the filter PADs. (**C**) Comparison among different concentrations of 4 μL of GOx pre-loaded on the filter PADs; the concentrations reported are referred to the final concentrations obtained when the sample solution is added to the origami sensor. A 40-μL-sample solution of 10 mM glucose was used as the sample solution in each case

The effect of the GOx pre-loading on the paper was studied by evaluating its enzymatic activity. In detail, two configurations have been designed by drop-casting a volume of 4 μL of 250 U/mL GOx (i) on the sampling area of the filter PAD or (ii) directly on the working area of the office paper-based sensor (Fig. 1A). After drying, the origami sensor was composed as follows: (i) in the first case, the GOx-PAD was overlapped onto the office paper CB/PBNP-modified sensor, and the measurement was carried out by adding glucose onto GOx-PAD; (ii) in the second case, a bare filter PAD was overlapped onto the office paper CB/PBNP-modified sensor on which GOx was previously loaded. The chronoamperometric measurements were thus carried out by drop-casting 40 μL of 10 mM glucose on the filter PAD of the folded GOx-origami biosensor. These responses were compared with the signal obtained using the origami sensor without pre-loading the enzyme, adding 40 μL solution containing both 10 mM glucose and 25 U/mL GOx (Fig. 1A). As expected, the chronoamperometric signals for GOx pre-loaded on the paper substrate were lower than the case using GOx in solution, in agreement with the literature [36]. The comparison between the filter paper and the office paper revealed that the current intensity obtained by pre-loading GOx on the filter PAD was almost double as compared with by pre-loading GOx on the office paper, which is ascribable to the higher porosity of the filter paper than the office paper. This evidence confirmed that the pre-loading of GOx on the filter PAD was suitable for the construction of a reagent-free origami sensor.

Subsequently, the volume for the GOx pre-loading on the filter PAD was investigated. Volumes equal to 2, 4, and 6 μL were used for the pre-loading step, keeping GOx concentration at 25 U/mL. Figure 1B shows that the chronoamperometric response slightly increases according to the increasing volume. However, the inset reported in Fig. 1B highlights that a 4 μL drop is the volume suitable to be homogeneously absorbed within the hydrophilic area of the filter PAD, while 2 μL drop leads to an uncomplete absorption. In the case of 6 μL, the higher volume was associated with a reduced repeatability. For these reasons, the volume of 4 μL was chosen for the pre-loading of GOx.

Finally, the concentration of GOx pre-loaded on the filter PAD was studied in the range between 1 and 25 U/mL, as shown in Fig. 1C. The chronoamperometric signal increases sharply up to 7.5 U/mL, after that only a slight increase is observed. The best compromise in terms of sensitivity and repeatability was obtained for 10 U/mL, which was hence selected.

### Configuration of the bienzymatic origami sensor

**Fig. 2:**
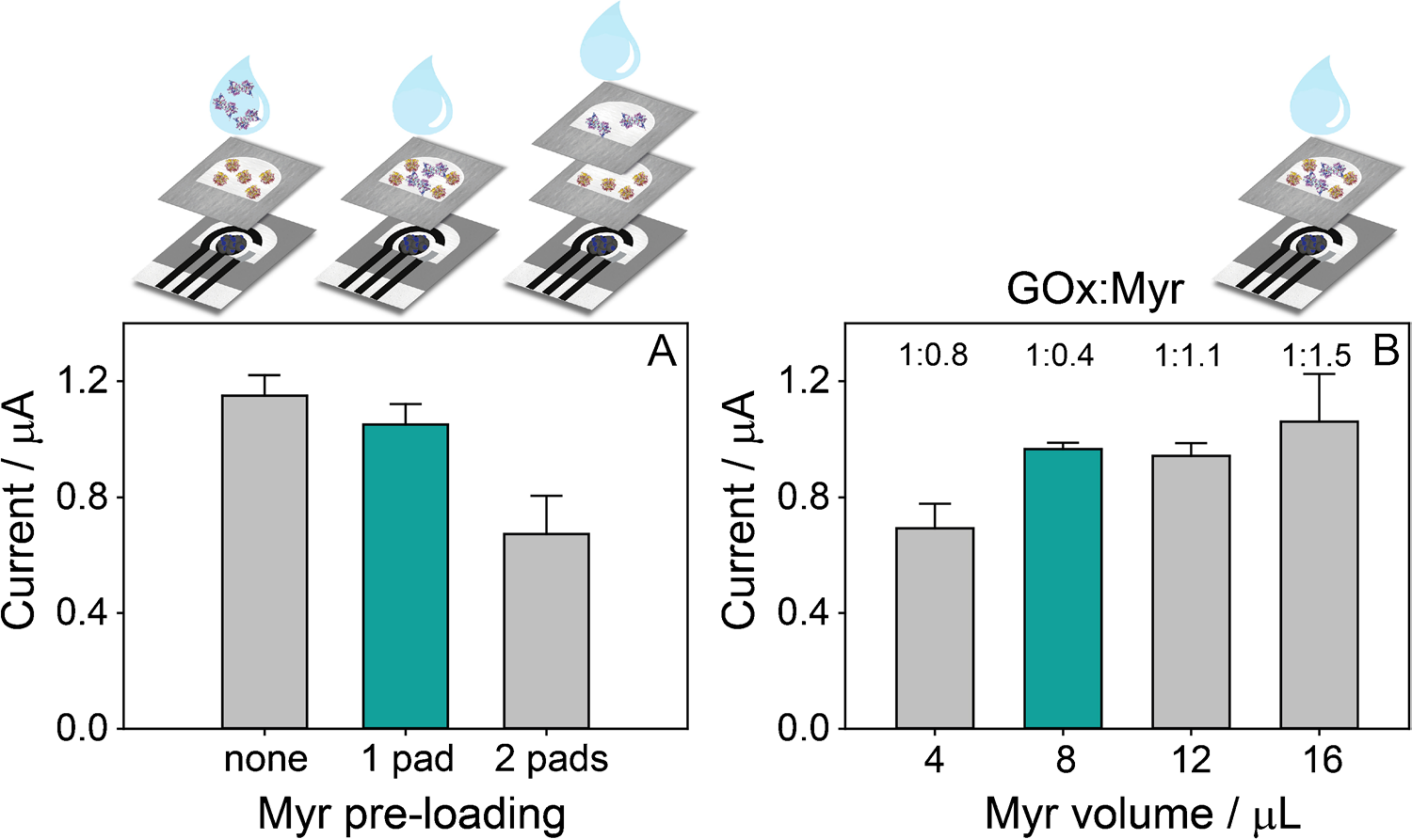
Chronoamperometric responses (*E* = 0.0 V, *t* = 300 s) obtained for the study of the pre-loading step of Myr. (**A**) Comparison among 8 μL of Myr pre-loaded on the filter PAD already containing GOx, 8 μL of Myr pre-loaded on a separated filter PAD, and Myr added in the sample solution; in all the cases, 38 U/mL Myr was used for the pre-loading, obtaining a final concentration of 7.6 U/mL when the sample solution was added. (**B**) Comparison among different volumes used to pre-load 38 U/mL Myr on a single filter PAD already containing GOx; the ratios reported are referred to the ratios between the final concentrations of GOx and Myr obtained when the sample solution is added on the origami sensor. A 40-μL-sample solution of 1 mM sinigrin was used as the sample solution in each case

Once selected the conditions for GOx pre-loading, Myr was introduced in the configuration of the origami device to realize the bienzymatic platform. In detail, two possible configurations were tested: (i) the use of a single filter PAD for the pre-loading of both enzymes and (ii) the use of two filter PADs where GOx and Myr were separately pre-loaded. For both strategies, 4 μL of 100 U/mL GOx and 8 μL of 38 U/mL Myr were used for the pre-loading step, and a drop of 40 μL of a standard solution of 1 mM sinigrin was placed on the origami biosensor, after overlapping the paper layers (Fig. 2A). For comparison, an origami biosensor with only GOx pre-loaded was tested by drop-casting 40 μL of a solution containing 1 mM sinigrin and 7.6 U/mL Myr. The responses obtained by using these configurations are reported in Fig. 2A. The signal obtained when Myr is added to the sample solution is slightly higher than the signal recorded for Myr pre-loaded on the same PAD. When comparing the use of a single filter PAD or two PADs for the separate pre-loading of the enzymes, a clear improvement of the chronoamperometric signal is observed for the single filter PAD. It can be speculated that the presence of a double layer of filter paper can hinder the diffusion of the enzyme substrates through the origami device, thus decreasing the amount of H_2_O_2_ by-product reaching the electrode surface. Consequently, the use of a single filter PAD was chosen to complete the configuration of the bienzymatic origami biosensor.

Finally, the amount of Myr to be pre-loaded on the single filter PAD was studied. Indeed, the GOx:Myr enzyme ratio is a key factor, due to the sequential reactions that occur between these two enzymes. To obtain different concentrations of Myr, increasing volumes of Myr 38 U/mL were pre-loaded on the filter PAD, namely 4, 8, 12, and 16 μL, corresponding to a GOx:Myr concentration ratio of about 1:0.8, 1:0.4, 1:1.1, and 1:1.5, respectively. As reported in Fig. 2B, the volume of 8 μL, corresponding to a final concentration of 7.6 U/mL, gives the best compromise between current intensity and repeatability, and it was thus selected for the analysis of real samples.

### Analytical performances

**Fig. 3:**
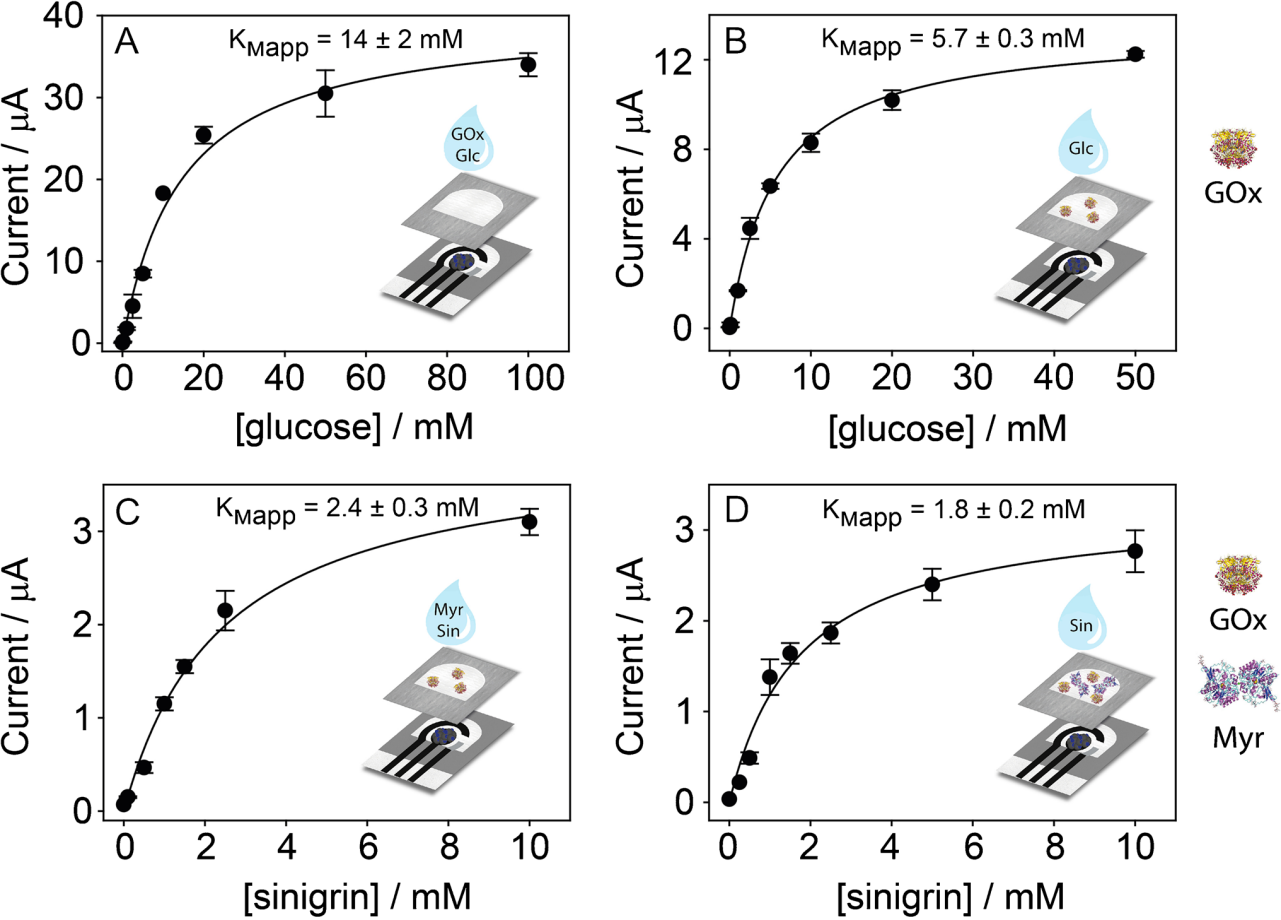
Michaelis–Menten curves obtained by chronoamperometry (*E* = 0.0 V, *t* = 300 s) for different configurations of the origami (bio)sensors: (**A**) 40 μL of a solution containing GOx and increasing concentrations of glucose were added to the origami sensor; (**B**) 40 μL of a solution containing increasing concentrations of glucose was added to the origami monoenzymatic biosensor, with GOx pre-loaded on the filter PAD; (**C**) 40 μL of a solution containing Myr and increasing concentrations of sinigrin were added on the origami monoenzymatic biosensor, with GOx pre-loaded on the filter PAD; (**D**) 40 μL of a solution containing increasing concentrations of sinigrin were added to the origami bienzymatic biosensor, with both GOx and Myr pre-loaded on the filter PAD. In all the cases, the final concentrations of GOx and Myr were 10 U/mL and 7.6 U/mL, respectively

The selected conditions for the preparation of the paper-based origami platform were applied to study the GOx activity for the monoenzymatic biosensor and the Myr/GOx reactions for the bienzymatic biosensor. In detail, the activity of each enzyme was tested for increasing concentrations of its substrate (i.e., glucose or sinigrin), by comparing the enzyme pre-loaded on the filter PAD or the enzyme added in the sample solution. In Fig. 3, the resulting curves and the fit applied to calculate the apparent Michaelis–Menten constants (*K*_Mapp_) are reported. In the case of the monoenzymatic biosensor (Fig. 3A, B), a significant decrease of *K*_Mapp_ was observed when the GOx was previously loaded on the filter PAD. This evidence can be ascribed to the porous nature of the filter paper, which allows for a homogeneous distribution of the enzyme within its matrix and provides a local environment suitable for the efficient reaction between the enzyme and its substrate. Coherently, a slight decrease was obtained also for the *K*_Mapp_ calculated in the case of the bienzymatic biosensor when Myr was pre-loaded on the filter PAD (Fig. 3C, D). The analytical performance corresponding to the final configurations chosen for the monoenzymatic biosensor (Fig. 3B) and the bienzymatic biosensor (Fig. 3D) is described in Table 1, with limits of detection (LOD) calculated using the ratio Signal/Noise = 3. The repeatability using the biosensing platform with Myr/GOx pre-loaded on the PADs was assessed by testing in triplicate 10 mM of glucose and 1 mM of sinigrin, obtaining an RSD% equal to 5% and 10%, respectively.

**Table 1:** Analytical features of the monoenzymatic and the bienzymatic origami biosensors in the buffer medium and extracted samples from Indian mustard seeds, upon additions of standard sinigrin or standard glucose. Buffer medium: 50 mM phosphate buffer + 50 mM KCl, pH = 6; the extracts were analyzed under the same buffer conditions, obtained by adding the minimum amount of a concentrated buffer solution

Analytical features	Monoenzymatic biosensor (glucose detection)	Bienzymatic biosensor (sinigrin detection)
Equation in buffer medium *y* = (*m* ± *σ*_m_)*x* + (*y*_0_ ± *σ*_*y*0_)	*y* = (1.86 ± 0.07)*x* + (− 0.01 ± 0.09)	*y* = (1.15 ± 0.07)*x* + (− 0.03 ± 0.06)
Linear range in buffer medium / LOD	0.15–2.5 mM / 0.05 mM	0.25–1.5 mM / 0.07 mM
Equation in extracted sample *y* = (*m* ± *σ*_m_)*x* + (*y*_0_ ± *σ*_*y*0_)	*y* = (1.04 ± 0.06)*x* + (0.17 ± 0.02)	*y* = (1.53 ± 0.07)*x* + (0.37 ± 0.02)
		Extracts diluted 1:2 v/v *y* = (1.7 ± 0.2)*x* + (0.19 ± 0.08)
Linear range in extracted sample	Up to 0.5 mM	Up to 0.5 mM
Concentration extrapolated	0.17 ± 0.02 mM	0.24 ± 0.03 mM
		Extracts diluted 1:2 v/v 0.22 ± 0.02 mM

In Table 2, a comparison with other examples of electrochemical biosensors for glucosinolate detection described in the literature is reported. It can be observed that the developed device reports a linear range in a higher concentration level (i.e., millimolar level) with respect to some of the examples from the literature (i.e., micromolar level), and consequently a higher LOD. However, this device is the first example of a paper-based biosensor in an origami configuration that offers the advantages of easiness and readiness-to-use (i.e., it requires just dropping the sample on the origami biosensor) as well as the multiplex analysis of both glucose and glucosinolates (i.e., sinigrin) with a single platform.

**Table 2:** Comparison among the analytical features of different bienzymatic (bio)sensors for glucosinolate detection

Bienzymatic sensor type	Method	Analyte(s)	Linear range	LOD	Real matrix	Ref
Myr-GOx on a platinized carbon base electrode oxygen electrode cell	Chronoamperometry	Sinigrin Progoitrin	Up to 5 mM	Not reported	Rape seeds	[30]
Myr-GOx on eggshell membrane placed on an oxygen electrode	PASCO oxygen sensor	Sinigrin	25–750 μM	Not reported	Cabbage Rape Mustard Caixin	[45]
Myr-GOx on eggshell membrane placed on an oxygen/optical biosensor	Fluorescence spectroscopy	Sinigrin	Glucose: about 1–2 mM	Not reported	Watercress Choi sum Kai choi Spinach	[12]
Myr-GOx-colloidal Gold-MWCNTs composite electrode	Chronoamperometry	Sinigrin	0.02–1 mM	5.9 μM	Brussel sprouts seeds	[11]
Myr-GOx on filter pad placed on CB-PBNP-modified office paper sensor	Chronoamperometry	Sinigrin Glucose	Glucose: 0.15–2.5 mM Sinigrin: 0.25–1.5 mM	Glucose: 0.05 mM Sinigrin: 0.07 mM	Indian mustard seeds	This work

*Myr* myrosinase, *GOx* glucose oxidase, *MWCNTs* multi-walled carbon nanotubes, *CB-PBNPs* Carbon black-Prussian Blue nanoparticles

### Real sample analysis

**Fig. 4:**
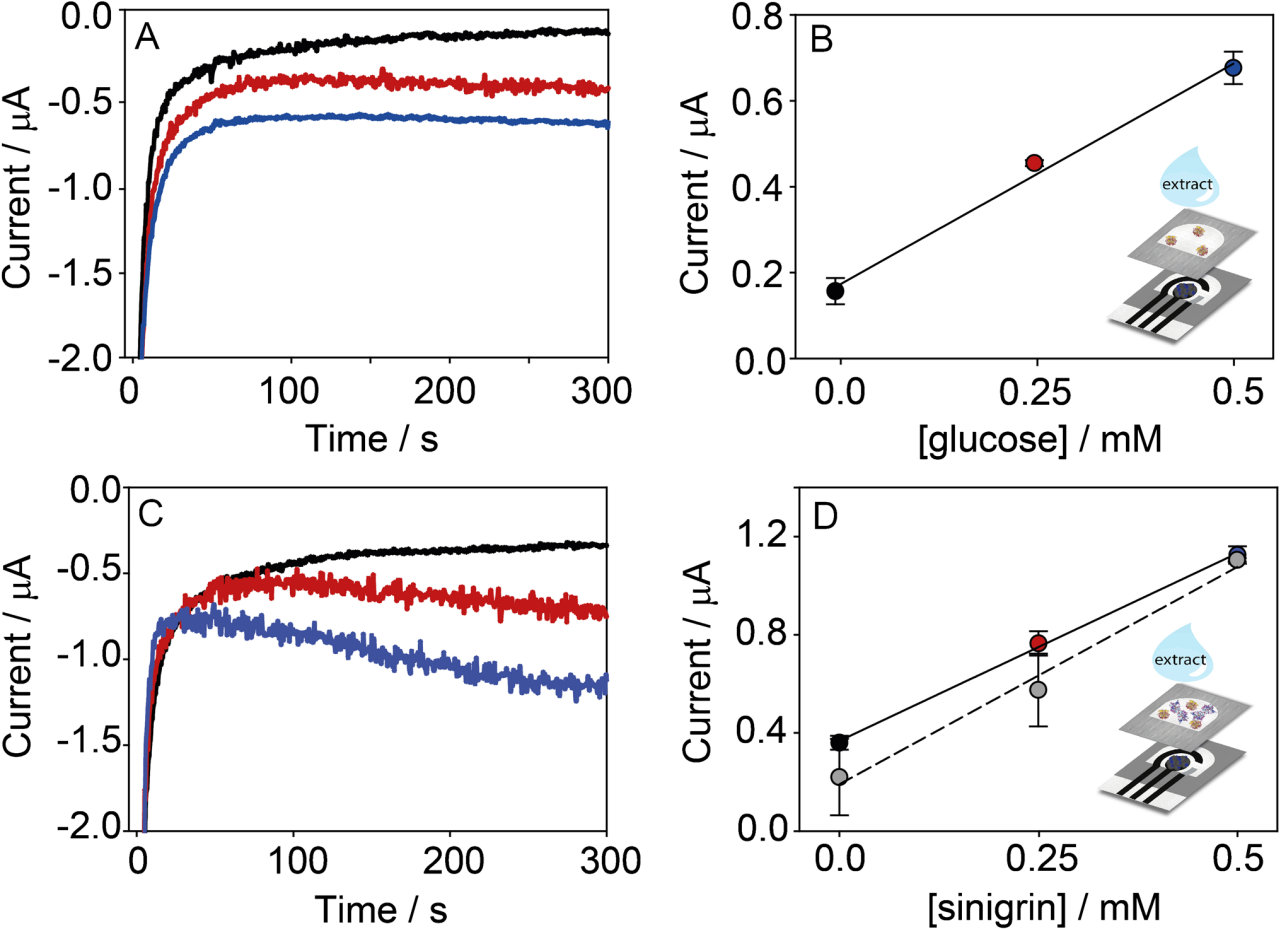
Chronoamperometric responses (*E* = 0.0 V, *t* = 300 s) and the corresponding plots obtained for the additions of standard solutions of glucose (**A**, **B**) and sinigrin (**C**, **D**) in the extracted samples from Indian mustard seeds. The measurements were carried out using the monoenzymatic origami biosensor (**A**, **B**) and the bienzymatic origami biosensor (**C**, **D**) by adding 40 μL of the extracted sample on the assembled origami. Current values are reported in the plots as absolute values (**B**, **D**). The data reported in gray with the dashed line refer to the extracted samples from Indian mustard seeds after dilution 1:2 v/v with the buffer solution

The response of the paper-based origami platform in real samples was studied in water extracts obtained from Indian mustard seeds by boiling. In detail, 0.1 g of sample was treated as described in “Experimental”, according to the literature [41]. To evaluate the analytical response of our paper-based origami platform in this matrix, the addition method was employed by adding known amounts of both sinigrin and glucose to obtain concentrations equal to 0.25 and 0.5 mM (Fig. 4). Table 1 reports the equations of the responses shown in Fig. 4, compared with the equations of the linear range obtained in standard solutions. The original concentrations of sinigrin and glucose in the extracted samples were extrapolated using the corresponding equations, showing that a significant amount of these analytes is detectable in these samples (Table 1). A loss of a linear response was observed for standard sinigrin higher than 0.5 mM, which can be ascribed to an overload effect due to the presence of endogenous sinigrin and glucose. To quantify sinigrin without overestimation, due to the presence of endogenous glucose, the current intensity recorded for glucose was subtracted to the current intensity observed for sinigrin in the sample extract; in this way, the amount of sinigrin calculated is equal to 0.07 ± 0.02 mM.

It is worthy to note that no additional treatments of the real matrix were required. Indeed, the application of the standard addition methods (i.e., 25 mM and 0.5 mM of sinigrin standard) to the extracted samples upon dilution 1:2 v/v with the buffer solution resulted in calculated values of sinigrin consistent with the extracts not diluted (see Fig. 4D and data in Table 1). As widely reported in the literature [33, 34], indeed, the filtering capability of the use of the porous filter PADs can significantly reduce the matrix effect. This evidence highlights how the properties of paper can play multiple roles at the same time, being a suitable material for the loading of the needed biocomponents (i.e., here Myr and GOx enzymes) while also preventing eventual interfering phenomena in a real complex matrix.

Once having proved the efficient performance of both the monoenzymatic and the bienzymatic origami biosensors in the extracted samples, a recovery study was carried out. In detail, known amounts of standard sinigrin were added upstream the extraction process for 0.1 g of Indian mustard seeds (see paragraph “Real sample extraction”), to obtain concentrations equal to 0.25 and 0.5 mM. The standard addition method was applied (i.e., by adding 0.25 and 0.5 mM of sinigrin standard solutions) to the undiluted extracts. The resulting chronoamperometric response showed good recovery values equal to (111 ± 3) % and (86 ± 1) % for 0.25 mM and 0.5 mM, respectively.

## Conclusions

Origami paper-based devices have established a new route in sustainable analytical tools due to their easy assembling, rending them a cost-effective, plastic-free, and miniaturized laboratory in a paper strip. Indeed, the integration by a simple overlapping of filter paper PADs, in which the reagents are loaded, and an office paper layer, in which the electrodes are printed, allows for a reagent-free measurement without asking the end-users any additional task, i.e., the addition of further reagents to carry out the measurement. In this work, we exploited these features to design a multiplex analysis for quality control of composition values in *Brassicaceae* plants, by detecting both glucose and glucosinolate. The use of different PADs, in which glucose oxidase or glucose oxidase/myrosinase are pre-loaded in the cellulose matrix, allows for the detection of two different analytes by using the same type of electrochemical sensor, printed on office paper. The hydrogen peroxide, which is the enzymatic by-product of the enzymatic reactions involved, was detected at low applied potential (i.e., 0 versus Ag/AgCl pseudoreference) by using an office paper printed electrochemical sensor modified with a nanocomposite constituted of Carbon black and Prussian Blue nanoparticles. The advantages of the developed multianalyte paper-based platform relies on the cost-effectiveness combined the easiness of applicability (i.e., ready-to-use, thanks to the enzyme pre-loading in the filter PADs), and the easiness of disposal (e.g., incineration), overall resulting in a highly sustainable approach. However, the limited linear ranges observed suggest that this device can be employed as a fast and easy-to-use screening method for glucosinolate content in *Brassicaceae* samples. The results obtained in real samples demonstrated that our paper-based platform is suitable for the detection of both glucose and sinigrin analytes in extracts from mustard seeds. Thanks to the versatility of these paper-based sustainable devices, it is intriguing to note that the multianalyte paper-based platform can be the starting point for the designing of additional origami paper-based (bio)sensors to the platform, extending the concept of multiplex detection in a single sustainable and easy-to-use device.

## References

[1] Pfaltzgraff LA, De Bruyn M, Cooper EC (2013). Food waste biomass: a resource for high-value chemicals. Green Chem.

[2] Pacifico D, Lanzanova C, Pagnotta E (2021). Sustainable Use of Bioactive Compounds from Solanum tuberosum and Brassicaceae wastes and by-products for crop protection-a review. Molecules.

[3] Russian Agricultural Sciences. In: Springer. https://www.springer.com/journal/11978. Accessed 3 Mar 2022

[4] Stojceska V, Ainsworth P, Plunkett A (2008). Cauliflower by-products as a new source of dietary fibre, antioxidants and proteins in cereal based ready-to-eat expanded snacks. J Food Eng.

[5] Engels C, Schieber A, Gänzle MG (2012). Sinapic acid derivatives in defatted Oriental mustard (Brassica juncea L.) seed meal extracts using UHPLC-DAD-ESI-MSn and identification of compounds with antibacterial activity. Eur Food Res Technol.

[6] Almuhayawi MS, AbdElgawad H, Al Jaouni SK (2020). Elevated CO_2_ improves glucosinolate metabolism and stimulates anticancer and anti-inflammatory properties of broccoli sprouts. Food Chem.

[7] Hu Y, Liang H, Yuan Q, Hong Y (2010). Determination of glucosinolates in 19 Chinese medicinal plants with spectrophotometry and high-pressure liquid chromatography. Nat Prod Res.

[8] Piekarska A, Kusznierewicz B, Meller M (2013). Myrosinase activity in different plant samples; optimisation of measurement conditions for spectrophotometric and pH-stat methods. Ind Crops Prod.

[9] Krul C, Humblot C, Philippe C (2002). Metabolism of sinigrin (2-propenyl glucosinolate) by the human colonic microflora in a dynamic in vitro large-intestinal model. Carcinogenesis.

[10] Gallaher CM, Gallaher DD, Peterson S (2012). Development and validation of a spectrophotometric method for quantification of total glucosinolates in cruciferous vegetables. J Agric Food Chem.

[11] Serafín V, Agüí L, Yáñez-Sedeño P, Pingarrón JM (2009). Glucosinolate amperometric bienzyme biosensor based on carbon nanotubes-gold nanoparticles composite electrodes. Electroanalysis.

[12] Choi MMF, Liang MMK, Lee AWM (2005). A biosensing method with enzyme-immobilized eggshell membranes for determination of total glucosinolates in vegetables. Enzyme Microb Technol.

[13] Aires A, Mota VR, Saavedra MJ (2009). Initial in vitro evaluations of the antibacterial activities of glucosinolate enzymatic hydrolysis products against plant pathogenic bacteria. J Appl Microbiol.

[14] Jing B, Guo R, Wang M (2020). Influence of seed roasting on the quality of glucosinolate content and flavor in virgin rapeseed oil. LWT.

[15] Ishikawa S, Maruyama A, Yamamoto Y, Hara S (2014). Extraction and characterization of glucosinolates and isothiocyanates from rape seed meal. J Oleo Sci.

[16] Szmigielska AM, Schoenau JJ, Levers V (2000). Determination of glucosinolates in canola seeds using anion exchange membrane extraction combined with the high-pressure liquid chromatography detection. J Agric Food Chem.

[17] Miklavčič Višnjevec A, Tamayo Tenorio A, Steenkjær Hastrup AC (2021). Glucosinolates and isothiocyantes in processed rapeseed determined by HPLC-DAD-qTOF. Plants.

[18] Shi Y, Zheng C, Li J (2018). Separation and quantification of four main chiral glucosinolates in Radix isatidis and its granules using high-performance liquid chromatography/diode array detector coupled with circular dichroism detection. Molecules.

[19] Chowdhury M, Ngo VD, Islam MN (2021). Estimation of glucosinolates and anthocyanins in kale leaves grown in a plant factory using spectral reflectance. Horticulturae.

[20] Kim SY, Seo HY, Ha JH (2020). A colorimetric sensor array for the discrimination of glucosinolates. Food Chem.

[21] Toledo-Martín EM, Font R, Obregón-Cano S (2017). Rapid and cost-effective quantification of glucosinolates and total phenolic content in rocket leaves by visible/near-infrared spectroscopy. Molecules.

[22] Cinti S, Cusenza R, Moscone D, Arduini F (2018). Paper-based synthesis of Prussian Blue nanoparticles for the development of whole blood glucose electrochemical biosensor. Talanta.

[23] Pagkali V, Soulis D, Kokkinos C, Economou A (2022) Fully drawn electrochemical paper-based glucose biosensors fabricated by a high-throughput dual-step pen-on-paper approach with commercial writing stationery. Sensors and Actuators B Chemical 358- 131546.10.1016/j.snb.2022.131546

[24] Mandpe P, Prabhakar B, Gupta H, Shende P (2020). Glucose oxidase-based biosensor for glucose detection from biological fluids. Sens Rev.

[25] Saravanan J, Pannipara M, Al-Sehemi AG (2021). Flower-like CuO/NiO nanostructures decorated activated carbon nanofiber membranes for flexible, sensitive, and selective enzyme-free glucose detection. J Mater Sci: Mater Electron.

[26] Kawde AN, Aziz MA, El-Zohri M (2017). Cathodized gold nanoparticle-modified graphite pencil electrode for non-enzymatic sensitive voltammetric detection of glucose. Electroanalysis.

[27] Shakir S, Saravanan J, Rizan N (2018). Fabrication of capillary force induced DNA template Ag nanopatterns for sensitive and selective enzyme-free glucose sensors. Sens Actuators, B Chem.

[28] Rani SD, Ramachandran R, Sheet S (2020). NiMoO_4_ nanoparticles decorated carbon nanofiber membranes for the flexible and high performance glucose sensors. Sens Actuators, B Chem.

[29] Saravanan J, Vignesh A, Shah SS (2022). Binder-less and free-standing Co–Fe metal nanoparticles-decorated PVdF-HFP nanofiber membrane as an electrochemical probe for enzyme-less glucose sensors. Res Chem Intermed.

[30] Koshy A, Bennetto HP, Delaney GM (1988). An enzyme biosensor for rapid assay of glucosinolates. Anal Lett.

[31] Leoni O, Iori R, Palmieri S (1991). Immobilization of myrosinase on membrane for determining the glucosinolate content of cruciferous material. J Agrie FoodChem.

[32] Tsiafoulis CG, Prodromidis MI, Karayannis MI (2003). Development of a flow amperometric enzymatic method for the determination of total glucosinolates in real samples. Anal Chem.

[33] Colozza N, Caratelli V, Moscone D, Arduini F (2021). Paper-based devices as new smart analytical tools for sustainable detection of environmental pollutants. Case Stud Chem Environ Eng.

[34] Colozza N, Caratelli V, Moscone D, Arduini F (2021). Origami paper-based electrochemical (Bio)sensors: state of the art and perspective. Biosensors.

[35] Colozza N, Kehe K, Popp T (2021). Paper-based electrochemical sensor for on-site detection of the sulphur mustard. Environ Sci Pollut Res Int.

[36] Colozza N, Kehe K, Dionisi G (2019). A wearable origami-like paper-based electrochemical biosensor for sulfur mustard detection. Biosens Bioelectron.

[37] Arduini F, Cinti S, Caratelli V (2019). Origami multiple paper-based electrochemical biosensors for pesticide detection. Biosens Bioelectron.

[38] Caratelli V, Fegatelli G, Moscone D, Arduini F (2022). A paper-based electrochemical device for the detection of pesticides in aerosol phase inspired by nature: a flower-like origami biosensor for precision agriculture. Biosens Bioelectron.

[39] Caratelli V, Ciampaglia A, Guiducci J (2020). Precision medicine in Alzheimer’s disease: an origami paper-based electrochemical device for cholinesterase inhibitors. Biosens Bioelectron.

[40] Cinti S, Arduini F, Vellucci G (2014). Carbon black assisted tailoring of Prussian Blue nanoparticles to tune sensitivity and detection limit towards H_2_O_2_ by using screen-printed electrode. Electrochem Commun.

[41] Doheny-Adams T, Redeker K, Kittipol V (2017). Development of an efficient glucosinolate extraction method. Plant Methods.

[42] Cinti S, Moscone D, Arduini F (2019). Preparation of paper-based devices for reagentless electrochemical (bio)sensor strips. Nat Protoc.

[43] Divakaran M, Babu KN, Caballero B, Finglas PM, Toldrá F (2016). Mustard. Encyclopedia of food and health.

[44] Cinti S, Arduini F, Moscone D (2014). Development of a hydrogen peroxide sensor based on screen-printed electrodes modified with inkjet-printed Prussian Blue nanoparticles. Sensors.

[45] Wu B, Zhang G, Shuang S (2005). A biosensor with myrosinase and glucose oxidase bienzyme system for determination of glucosinolates in seeds of commonly consumed vegetables. Sens Actuators B Chem.

